# Populism Is Always Gendered and Dangerous

**DOI:** 10.3389/fsoc.2020.625385

**Published:** 2021-01-11

**Authors:** Julie Mostov

**Affiliations:** New York University, New York City, NY, United States

**Keywords:** gender, patriarchy, populism, nationalism, democracy

## Abstract

This article argues that populism is always gendered and dangerous to women and democracy. The distinctive reliance on the polarization of “us” and “them” in populism draws on nationalist notions of exclusive belonging, the need for closure to protect the “us” from would be infiltrators, and observance of proscribed gendered roles to ensure the continued rule of the majority (race/ethno-nation). The reproduction of the “us” is too crucial to leave unregulated, and gendered bodies are too vulnerable to violation and occupation to go without vigilance, that is, without surveillance and demographic policing. Gendered narratives support the anti-immigration features of populism and its curbs on democratic institutions, both in the service of national recovery and in its identification of potentially disloyal, suspect voices within the demos.

Populism values the crowd over engaged citizens. Populist leaders who stoke the enthusiasm of the crowd rely on an identification of sameness among its members and incite suspicion of those who might question the message, who might not be properly loyal or who might be susceptible to the “contamination” of others. Even if the energy is focused on tearing down the establishment and weeding out corruption or systems of exploitation, the distinctive nature of reliance on “us” v. “them” always carries the seeds of a kind of exclusive belonging and the perilous path of proscribed gendered roles. The reproduction of the “us” is too crucial to leave unregulated, and gendered bodies are too vulnerable to violation and occupation to go without vigilance, that is, without surveillance and demographic policing. Proper gender roles are essential to the nationalist narratives of populist imaginaries, fueled by resentment at the demeaning roles left to beleaguered members of the “should-be dominant nation” by condescending elites or corrupt politicians. These gendered roles are often noted in feminist critiques of right-wing nationalism, but the relationship between gender and populism is understudied in the increasingly pervasive literature on populism (Abi-Hassan, 2017). In some cases, theorists question the specifically gendered nature of populism (as opposed to particular instantiations of it). Others, while recognizing the hypermasculinity of populist leaders and their rhetoric, qualify this by noting the currently increasing number of women engaged in or among the leadership of populist parties (Farris, 2017; Kantola and Lombardo, 2019).

In this short piece, my major focus is not to address the wide-ranging literature on populism, nor to explore the absence of gender from important discussions of populism, but to make the argument that populism is always gendered and dangerous to women and democracy.

While contested in terms of right and left associations and historical relations to fascism or democracy, theorists generally associate populism with the energized “ordinary” people who have been ignored and demeaned by an elitist few who have gained control of society in their own interests (often by bringing in or racial, religious, or ethnic others/minorities) at the cost of the people and, even, the Nation. The “authentic” people rally under a charismatic leader who claims to be in a unique position to define and defend the interests of the people and to recover the values which support the people's prominence in the nation and restore the Nation to its glorious past (Cohen, 2019; Urbinati, 2019). Anti-immigration policies and religious or ethnic bigotry, thus, often play important roles in the rhetoric of populists, together with an imaginary of violation, occupation, and displacement by the dangerous, criminalized, and devious others threatening the “assumed” majority, its rule and social, economic, and political standing (Beltrán, 2020). I do not associate these characteristics of populism with popular leaders, such as Bernie Sanders, who energize voters around critiques of inequality and social injustice, as well as notions of solidarity and recognition of difference. It is when popular movements eschew these principles and embrace exclusionary practices that they edge toward populism and its dangers.

Populisms thrive on the devotion to sameness in the protection of “us” against the dangerous others and those “elites” who are ready to sacrifice us for them. This may speak to unemployed workers against immigrants; women fearful of losing privileged protections; members of ethnonational, racial or religious groups who are pitted against one another for the minimal benefits of inclusion; privileged communities afraid of the “lawlessness” of those demanding their basic rights; and members of self-proclaimed religious majorities threatened by those who believe differently. Populism and nationalism are tightly linked and, while appearing benign in some cases, always are eventually dangerous for gender differences, for women, and democracy^1^.

The desire to naturalize national boundaries and ethnic/racial differences makes recourse to gendered roles in the reproduction of the nation particularly effective (Stevens, 1999; Mostov, 2008). As women have a special duty to reproduce the nation and ward off the threat of demographic tragedy, control of women's sexuality is tightly linked to control of national space and the transgressing of symbolic and physical borders. Women's role in reproducing the nation and upholding its values makes them also the object of suspicion and surveillance: “our” women can be seduced by the dangerous male other or refuse their proper roles by failing to reproduce or doing so willingly with the other; the “other's” women threaten the demographic balance by increasing their own numbers. This narrative may be obscured in populist party appeals to women's equality in the workplace or at the ballot box, even in party politics, but the demographic threat of diminishing racial or ethnic group numbers and standing sneaks its way back in more or less vocal ways into populist policies. Gendered bodies become the object of control, from direct attacks on abortion rights, to racialized reproductive incentives and patriarchal narratives that stigmatize the other's gendered practices, to family separations and decisions around deserving asylum seekers.

In gendered narratives of the nation, the other's males are depicted as either hyper sexualized predators of “our” women or impotent or “effeminate” failures, unable to protect “their” women or fulfill other accepted male roles (Mostov, 2008; Anand, 2011). At the same time, male migrants who risk life and limb for their families are characterized as deeply embedded in patriarchal cultures and, thus, likely to be abusive toward their “submissive” women. The only migrants deemed “deserving” of possible asylum are those women whose situation is so dire that they are at the mercy of the receiving hosts, who look to “save” them from both their men and their culture. This sets up relationships that continue to build inequality and discrimination into contemporary society. “Their” women remain a collective symbol of backwardness to be saved through possible assimilation (and incorporation into the economy—often in the private sector or care chain.) Their men remain collective threats to the “us” through their entry into the labor market, dilution of the majority culture, and threat to “our” safety as criminals, rapists, and terrorists, as well as threats to their own women. Cases of domestic violence or gender-based violence among the majority nation are rarely talked about as examples of cultural norms, but as individual cases of unfortunate behavior (Mostov, 1994; Abu-Lughod, 2002). The simplistic dichotomy of “us” vs. “them” in populist politics aligns with these gendered narratives and reaffirms the ways in which populist parties may recognize the protection of women's (or even gay) rights in their public denigration of the masculine other while ignoring these rights, even undermining their realization in everyday life (Farris, 2017).

Populists like Trump, Orban, Salvini, and Modi gain the attention of their adoring crowds by promising to restrict who is eligible to be part of the demos (for example, Hungary's acceptance of only Christian immigrants or India's restriction of Muslims' citizenship rights), and emboldening their followers with narratives of return to a once great homogenous nation of like-minded men (Löffler et al., 2020). It is only in a polity of such men that ordinary people can count on the outcomes of voting to support their privilege (even minimal as it is over others), their expectations of recognition, and control over their lives as breadwinners and heads of households. This masculine imaginary is embodied in the leaders' financial and personal success, rhetorical style and performative practices. This imaginary resonates with men by affirming their own gendered sense of self in the face of a reality that augers defeat, and by promising to expel those who have emasculated them. This masculine imaginary can appeal to women who gain a place at the table through it, who, themselves feel threatened in their precarious position as women (earlier denied the franchise and often questioned as capable of political judgement.) They, thus, may rise in their standing over racial or ethnic others by confirming their belonging to the would-be “dominant” nation. By virtue of this belonging, they demonstrate their fitness as part of the demos. But, it is still a position of precarity, entrenched at the cost to others.

Populists claim to be the bulwark against erosions of national pride by elites and others (traitors to the so-called majority race or ethno-nation/religion) who have reduced the standing of the ordinary citizens. Only when we can preserve our “national” character or our way of life from outsiders can we ensure the safety of democracy. In order to protect “our” democracy or desired political system, we need closure: closed borders, immigration restrictions, citizenship restrictions. We need to ensure that those allowed into the electorate are “fit.” Exclusions on immigration and to full citizenship are deeply entrenched in national histories. Attempts to protect the homogeneity of the demos or to define it in terms of ethnonational belonging and racial superiority or purity produce a range of exclusionary practices, including ethnic cleansing and deportation (Howard-Hassmann and Walsh-Roberts, 2015) as well as a constant questioning of the loyalty of those already within.

This linkage between closure and democracy has, in fact, fed the current slide to authoritarian populism. It brings the nationalist process of establishing external hard borders inside the polity, casting suspicion on “unacceptable” or dangerous members within the polity. It is accompanied by curbs on the free press, voter suppression, police surveillance and brutality, and challenges to the integrity of courts and other independent institutions. This link sets the stage for populist leaders (or what I call ethnocrats) to accumulate control over various public and private resources and maintain their positional gains through the absence of mechanisms for accountability. The wide spread loss of credible information from public offices and the ubiquity of the phrase “fake news” from Belgrade to Washington, DC, reflect this weakening of checks on abuses of power.

Thus, instead of the expected flourishing of democratic spaces (symbolized in the twentieth century by the fall of the Berlin Wall), we have been experiencing a de-democratization in the face of economic challenges from global capital, new cross-border technologies, and major political disruptions linked to shrinking arenas of state sovereignty (Mostov, 2008; Brown, 2010; Balibar, 2017). The call for more walls, technologies of surveillance, militarized campaigns against criminalized others, concerted efforts to stop the demographic decline of the self-proclaimed majority race or ethnic group, and expansion of borders within (and outside of) national spaces are all part of the populist trends to protect “us” and “our” national space.

We should be careful to distinguish popular social movements from such populist demands and populist political parties or movements. The rhetoric of closure against the menacing other is always a part of populism. Popular movements might reject compromised institutions and be suspicious of cooptation, but do not reject the solidarity of others, do not strive for an undiluted many—the power of the crowd—that has no place for the voice of difference. When those messages begin to emerge in popular movements the movements may splinter and change or lose their democratic appeal, become more or less exclusionary parties, and, in some cases lead to authoritarian regimes. Popular social movements can energize and enrich democracies, populism enervates them. When the crowd begins to see cracks in the “us” and looks for scapegoats for its demise, racial or ethnic others are there to blame. Populism organized as a binary of us and them, roots out potential “traitors.” Women are, invariably, among them; still indispensable to the reproduction of the nation, and always suspect in their loyalty to it.

## Author Contributions

The author confirms being the sole contributor of this work and has approved it for publication.

## Conflict of Interest

The author declares that the research was conducted in the absence of any commercial or financial relationships that could be construed as a potential conflict of interest.

## Footnotes

^1^Some regimes described as left-populisms (for example, Nicholas Maduro's Venezuela) may appear not to share the characteristics typical of right-wing nationalist populisms, but they do include elements such as the threat of internal and external enemies, the focus on loyalty, surveillance, and “us” vs. “them,” and a charismatic leader who rallies the crowd around the authentic interests of the people. These elements ultimately also make such populisms dangerous for gender differences, for women and democracy.
